# DNA damage—how and why we age?

**DOI:** 10.7554/eLife.62852

**Published:** 2021-01-29

**Authors:** Matt Yousefzadeh, Chathurika Henpita, Rajesh Vyas, Carolina Soto-Palma, Paul Robbins, Laura Niedernhofer

**Affiliations:** Institute on the Biology of Aging and Metabolism Department of Biochemistry, Molecular Biology and Biophysics, University of MinnesotaMinneapolisUnited States; University of RochesterUnited States; Weill Cornell MedicineUnited States

**Keywords:** DNA damage, DNA repair, Aging, progeria, genome instability

## Abstract

Aging is a complex process that results in loss of the ability to reattain homeostasis following stress, leading, thereby, to increased risk of morbidity and mortality. Many factors contribute to aging, such as the time-dependent accumulation of macromolecular damage, including DNA damage. The integrity of the nuclear genome is essential for cellular, tissue, and organismal health. DNA damage is a constant threat because nucleic acids are chemically unstable under physiological conditions and vulnerable to attack by endogenous and environmental factors. To combat this, all organisms possess highly conserved mechanisms to detect and repair DNA damage. Persistent DNA damage (genotoxic stress) triggers signaling cascades that drive cells into apoptosis or senescence to avoid replicating a damaged genome. The drawback is that these cancer avoidance mechanisms promote aging. Here, we review evidence that DNA damage plays a causal role in aging. We also provide evidence that genotoxic stress is linked to other cellular processes implicated as drivers of aging, including mitochondrial and metabolic dysfunction, altered proteostasis and inflammation. These links between damage to the genetic code and other pillars of aging support the notion that DNA damage could be the root of aging.

## Introduction

Aging is a multifactorial process that results in increased risk of a myriad of chronic diseases. Being elderly is the greatest risk factor, by orders of magnitude, for cancer, osteoporosis, cardiovascular disease, dementia and most other degenerative diseases (Kirkwood, 2005). While no single mechanism or pathway fully accounts for age-associated functional decline, one prevailing theory is that macromolecular damage, accumulating over time, plays a causal role in driving aging. Most macromolecules in the cell when damaged are simply degraded and replaced. In contrast, the nuclear genome, which is the blueprint for all cellular functions, has dedicated and energetically costly repair mechanisms to rapidly correct DNA damage. This intimates that DNA damage is a particularly hazardous type of macromolecular damage and therefore likely to be deleterious to cellular homeostasis.

Maintaining genome stability is a continuous process. Deoxyribonucleic acids are chemically unstable under physiological conditions (aqueous, oxygen-rich, and pH 7.4) (Lindahl, 1993). DNA is also vulnerable to chemical attack by electrophiles and free radicals. While exogenous sources of genotoxic stress can be quite potent, endogenous threats are constant and relentless (Table 1). The most common DNA lesion is hydrolytic cleavage of the glycosidic bond between the DNA base and sugar phosphate group, leading to abasic sites. Hydrolytic deamination of the DNA bases is also common. Products of normal cellular metabolism can cause oxidation, nitrosylation, and alkylation of the DNA bases (De Bont and van Larebeke, 2004). Breaks in the phosphate deoxyribose backbone arise as a consequence of high energy radiation or during DNA metabolism (replication, decatenation). Spontaneous DNA damage occurs on the order of 10^4^–10^5^ events per cell per day (Lindahl, 1993; De Bont and van Larebeke, 2004).

**Table 1.** Estimated frequencies of DNA lesions caused by endogenous and common environmental sources of DNA damage. Adapted from Friedberg, 2006; Lindahl, 1993; Sander et al., 2005; Sears and Turchi, 2012; Mouret et al., 2006.

**Endogenous DNA adducts**									
DNA lesion	DSB	Cytosine deamination	Cyclopurine adducts	Depyrimidination	8-oxoG	Malondialdehyde adducts	Alkylation adducts	Depurination	SSB
Frequency per cell per day	10^1^	10^2^	10^2^	10^2^	10^3^	10^3^	10^3^	10^4^	10^4^
**DNA adducts caused by environmental exposures**							
Genotoxin	Sunlight	Background radiation			Ionizing radiation therapy		Oxaliplatin cancer therapy
Lesion	Photodimers	Damaged bases	SSB	DSB	Damaged bases	SSB	Intra- and interstrand crosslinks
Frequency per cell per day	10^2^ in skin cells only	10	2–5	0.25	10^3^	10^3^	10^3^

DNA is also susceptible to damage by environmental factors such as ultraviolet (UV), ionizing radiation, and alkylating agents used to treat proliferative disorders like cancer (Table 1). Notably, even when exogenous genotoxin exposure is instigated with the purpose of driving cell death (e.g., in cancer therapy) adduct burdens are well below the incidence of endogenous damage (Jackson and Loeb, 2001). Fortunately, all organisms have robust mechanisms to sense all types of DNA damage, delay genome replication (if needed), signal for repair, and correct or tolerate the large number of genomic insults that occur on a daily basis (Hoeijmakers, 2009). DNA damage that is not repaired in a timely manner or is too egregious to be repaired induces signaling events that lead to one of many cell fates, one of which, senescence, plays a causal role in aging.

## Conceptually, could DNA damage drive aging?

Why? and how? organisms age remain challenging questions. Why one ages interrogates the reasons. How one ages interrogates the method. The antagonistic pleiotropic theory of aging provides a genetic solution to why we age, posing that genes that provide an advantage during reproductive life are disadvantageous post reproduction, yet these genes cannot be selected against (Kirkwood, 2005). As an example of antagonistic pleiotropy, activation of the DNA damage response (DDR) is critical for preventing cancer; however, chronic activation of the DDR is thought to drive the accumulation of senescent cells and chronic sterile inflammation in old age, as described in more detail below. The pillars of aging (Kennedy et al., 2014) describe the method by which (or how) we age: loss of or impaired, mitochondrial integrity and function, metabolism, stem cell function, proteostasis, nutrient sensing, adaptation to stress, autophagic flux, epigenetic control, and an accumulation of damaged cellular macromolecules. This includes damage to the nuclear and mitochondrial genomes. Nevertheless, it has proven challenging to establish the pillars of aging as true causes of aging rather than merely consequences of aging.

If DNA damage drives aging, mechanistically how does it do so? Through activating signaling responses (d'Adda di Fagagna et al., 2003), blocking transcription (Vermeij et al., 2016) and other DNA metabolism, altering the epigenome (Oberdoerffer et al., 2008), mutagenesis (Vijg, 2014), triggering cells senescence or apoptosis? DNA damage occurs stochastically but the amount and types of DNA damage one experiences is influenced by the expression of genes encoding antioxidant enzymes, genes linked to energetics and mitochondrial function, and a myriad of other factors such as histones, methylases, sirtuins, transcription, and replication factors. Every aspect of how DNA damage might drive aging is also genetically determined via the cellular response to DNA damage. The somewhat surprising finding is that DNA damage has far-reaching effects on many aspects of cellular metabolism tied to aging, the so-called pillars of aging (Kennedy et al., 2014). This suggests that aging might be driven by many types of cellular damage yet does not occur until one reaches a state where multiple aspects of cell biology are perturbed, for example, genome integrity, proteostasis, and mitochondrial function.

## DDR and cell fate decisions

**Figure 1.**
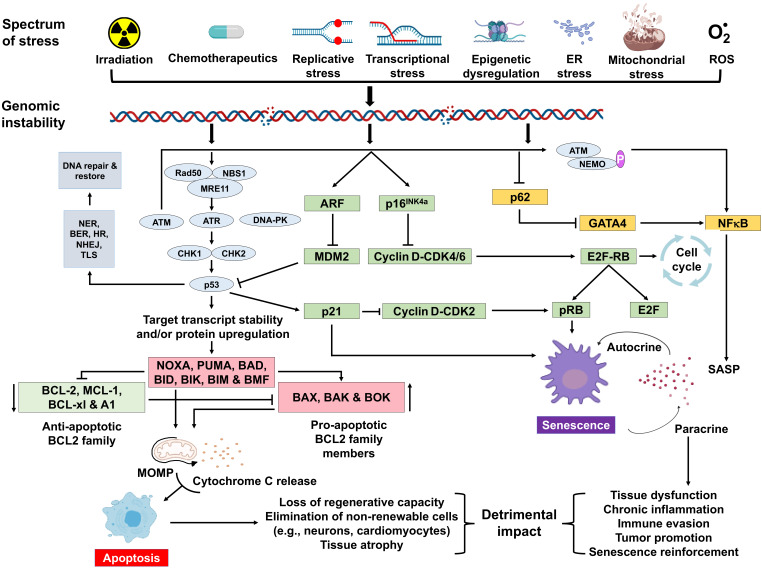
Schematic representation of signaling events within a cell that enable DNA damage to promote aging. Depicted are various stressors that can lead to genome instability and activation of the DNA damage response (DDR). The DDR (light blue) leads to cell cycle arrest (green). If signaling persists, apoptosis or senescence ensues. Senescence can affect neighboring, undamaged cells.

Once DNA damage is recognized in the nuclear genome, bulky adducts, small miscoding lesions, single-strand breaks, or non-complex double-strand breaks (DSBs) can be directly repaired by nucleotide excision repair (NER), base excision repair (BER), and non-homologous end-joining (NHEJ), respectively. If replication forks or transcription complexes encounter polymerase-blocking lesions (Vermeij et al., 2016), this can lead to the formation of a DSB or R-loop (Tresini et al., 2015), which potently activate signaling events that halt cell cycle progression and promote repair. If the damage signals persist, then the cell selects a fate that avoids replicating a damaged genome and mutagenesis at all cost, by activating events that lead to cell death (apoptosis) or irreversible cell cycle arrest (senescence) (Figure 1). DNA damage signaling begins with the MRE11-RAD50-NBS1 (MRN) complex activating the phosphatidylinositol 3-kinase-like kinases (PIKKs) ataxia-telangiectasia mutated (ATM), ATM-related kinase (ATR) and/or related PIKK (Thompson, 2012). ATM is activated primarily by DNA DSBs while ATR is primarily involved in the response to stalled replication forks, although overlap occurs. Initially ATM and ATR work with checkpoint mediator proteins like MDC1, 53BP1 and BRCA1, TOPBP1, which are sensor proteins that bind to lesions and recruit other DDR factors. Damage recognition is followed by phosphorylation (and activation) of transducer kinases like checkpoint kinase 2 (CHK2) or checkpoint kinase 1 (CHK1), which amplify the ATM-ATR signal. CHK2 activation leads to activation of p53, a master regulator of the cellular response to genotoxic stress (Senturk and Manfredi, 2013). Sustained activation of these transducer proteins leads to phosphorylation of several DNA repair and cell cycle checkpoint proteins (Thompson, 2012; Cheng and Chen, 2010). p53 can also be activated independently of ATM. p14/ARF inhibits MDM2–p53 interaction, resulting in the stabilization of p53 (Senturk and Manfredi, 2013; Meek and Anderson, 2009). p53 can mount a bimodal response to stress where it functions to activate apoptosis via various transcriptional targets like *Bax*, *Puma,* and *Noxa*, or promote transient or prolonged cell cycle arrest through transcriptional activation of the cyclin-dependent kinase inhibitor p21^CIP1^ (Reinhardt and Schumacher, 2012). A myriad of factors determines the extent of p53 activation in response to genotoxic stress, and more factors likely will be discovered.

A variety of types of stress can activate the *INK4a*/*ARF* locus in cells. The gene products of the locus—p16^INK4a^ and ARF—arrest cell progression by acting on the retinoblastoma protein and p53 (Senturk and Manfredi, 2013). p16^INK4a^ inhibits CDK4 from binding to cyclin D, thereby preventing phosphorylation of RB. Hypo-phosphorylated retinoblastoma (RB) protein represses E2F-dependent gene expression, blocking G1/S cell cycle progression. Free E2F activates many genes involved in mitosis. While sensing DNA damage and responding to it is a positive attribute, persistent DNA damage signaling can have quite deleterious effects. It can lead to chronic activation of p53 and other response pathways (stress response, pro- and anti-apoptotic, pro-inflammatory, etc.), which impact many aspects of cellular function and impact cell fate. Definitive knowledge about which factors determine the selection of cell fate selection remains elusive. However, it seems likely that these factors may vary with the cell type, the level, and the duration of genotoxic stress.

## Cellular senescence and the secretory phenotype

Leonard Hayflick and Paul Moorhead defined cellular senescence as the proliferative arrest that occurs in normal cells after a number of cell divisions (Hayflick and Moorhead, 1961; Hayflick, 1965). The first molecular explanation for senescence was the progressive telomere shorting that occurs with every cell division termed telomere-initiated cellular senescence (Harley et al., 1990). Many cells undergo senescence independently of telomere shorting due to replicative, mitochondrial, oxidative, metabolic, or genotoxic stress (Parrinello et al., 2003; Christoffersen et al., 2010; Correia-Melo et al., 2016; Nair et al., 2015). Senescent cells actively repress cell cycle progression preventing the replication of a damaged genome. Senescent cells have altered metabolism, morphology, and secretory profile called the senescence-associated secretory phenotype (SASP). The SASP includes pro-inflammatory cytokines, chemokines, and proteases (Coppé et al., 2008) and is heterogenous depending on the cell type and the inducer of senescence (Basisty et al., 2020). The SASP is thought to instigate immune clearance of the damaged/stressed cells (Tasdemir et al., 2016), but the immediate negative consequence is the potential to drive local tissue damage and systemic chronic sterile inflammation (Franceschi and Campisi, 2014).

One of the challenges of studying senescence is the absence of a specific marker to identify senescent cells. Therefore, multiple endpoints must be measured simultaneously to identify them (Gorgoulis et al., 2019; Sharpless and Sherr, 2015). The most widely used marker is measurement of senescence-associated β-galactosidase (SA-β-gal) activity (Itahana et al., 2007). Other markers associated with cellular senescence include absence of proliferation markers (e.g., Ki-67), increased expression of the cyclin-dependent kinase inhibitors p16^INK4a^ and p21^CIP1^, persistent activation of the DDR (such as p53, ATM, ATR, ɣH2AX, telomere dysfunction-induced foci [TIF], senescence-associated heterochromatin foci [SAHF], and DNA segments with chromatin alterations reinforcing senescence [DNA-SCARS]), reduced lamin B1 expression, increased HMGB1 and SASP factors (such as IL-1β, IL-6, IL-10, MCP-1, and TNFα) (Coppé et al., 2010; Lawless et al., 2010; Serrano et al., 1997; Takai et al., 2003; Matjusaitis et al., 2016; Rodier et al., 2011).

Cellular senescence plays a critical role in preventing tumorigenesis, tissue repair, and wound healing, promoting insulin secretion and mammalian development (Kuilman et al., 2008; Krizhanovsky et al., 2008; Demaria et al., 2014; Storer et al., 2013; Helman et al., 2016). However, time-dependent accumulation of senescent cells, likely through decreased immunoclearance, occurs in virtually all vertebrates (Jeyapalan et al., 2007; Liu et al., 2009) and has been definitively shown to drive aging (Baker et al., 2016), rather than simply occur with aging. Senescent cells also contribute to age-related diseases such as atherosclerosis (Roos et al., 2016), osteoporosis (Chandra et al., 2020), nonalcoholic fatty liver disease (Ogrodnik et al., 2017), cancer (Alimirah et al., 2020), neurodegenerative diseases (Chinta et al., 2018; Zhang et al., 2019), and numerous other age-related conditions (Kirkland and Tchkonia, 2020). Interestingly, NF-κB is the transcription factor that is most activated with aging (Adler et al., 2007). NF-κB signaling is the major signaling pathway that stimulates SASP (Tilstra et al., 2011). DNA damage activates NF-κB signaling via an ATM-NEMO-dependent regulation of an upstream kinase (Miyamoto, 2011; Figure 1). DNA damage can also lead to NF-κB activation via stabilization of GATA4 (Kang et al., 2015). Thus, genotoxic stress is a potent inducer of senescence and SASP, key drivers of aging and age-related disease.

## Accelerated aging in genome instability syndromes

The mammalian genome encodes over 150 proteins directly responsible for safeguarding its integrity (Friedberg, 2006; Wood et al., 2005). These gene products constantly monitor the quality and repair the nuclear genome (Sancar et al., 2004). Distinct DNA repair pathways cope with different types of DNA lesions: BER for small covalent additions to DNA bases, NER for bulky adducts that disrupt the DNA helix, interstrand crosslink (ICL) repair to remove covalent links between the two strands of DNA, NHEJ to ligate broken DNA ends, mismatch repair to correct replication errors, homologous recombination to manage replication stress and DSBs not readily ligated. Inherited defects in each of the DNA repair pathways are linked to distinct genome instability syndromes (Table 2). Broadly, the syndromes are characterized by developmental defects, increased incidence of cancer and features of accelerated aging (Cleaver, 1968; Menck and Munford, 2014; Wood, 2018). Progeroid syndromes are diseases of dramatically accelerated aging and include Hutchinson-Gilford, Werner, and Cockayne syndromes (CS), as well as XFE progeroid syndrome, all of which are linked to genome instability (de Magalhães, 2005; Martin and Oshima, 2000; Burla et al., 2018). The syndromes provide an elegant yet tragic glimpse into the impact that DNA damage can have on human health. Most syndromes were described well before DNA had been discovered or mechanisms of repair described.

**Table 2.** Human genome instability diseases with age-associated symptoms.

Disease	Affected genome stability pathway	Mutated genes	Aging-associated symptoms	Ref(s)
Hutchinson-Guilford progeria syndrome	Chromatin organization	*LMNA*	Alopecia, atherosclerosis, arthritis, cardiovascular disease, lipodystrophy, osteoporosis, skin aging and atrophy	Kudlow et al., 2007; Liu et al., 2005
Nestor-Guillermo progeria syndrome	Chromatin organization	*BANF1*	Alopecia, atherosclerosis, arthritis, cardiovascular disease, lipodystrophy, osteoporosis, and pulmonary hypertension	Cabanillas et al., 2011; Loi et al., 2016
Werner syndrome	Telomeric maintenance and replication stress	*WRN*	Alopecia, atherosclerosis, arthritis, cardiovascular disease, cataracts, diabetes, sarcopenia, and increased risk of cancer	Kudlow et al., 2007; Sugimoto, 2014
Rothmund-Thomson syndrome	DNA replication initiation	*RECQL4*	Alopecia, cataracts, osteoporosis, skin atrophy, and increased risk of cancer	Croteau et al., 2012; Ghosh et al., 2012
Bloom syndrome	DNA replication and recombination	*BLM*	Diabetes, pulmonary disease, increased risk of cancer	Hanada and Hickson, 2007; de Renty and Ellis, 2017
XFE progeroid syndrome	NER, ICL, and DSB repair	*ERCC4*	Anemia, cardiovascular disease, kidney disease, neurodegeneration, osteoporosis, sarcopenia, sensory loss, and skin atrophy	Niedernhofer et al., 2006
Xeroderma pigmentosum	NER and translesion DNA synthesis	*XPA-G*, *XPV*	Premature skin photoaging, neurodegeneration, and increased incidence of skin cancer	Lehmann et al., 2011; Kraemer and DiGiovanna, 2015
Cockayne syndrome	Transcription-coupled NER	*CSA*, *CSB*, *XPB*, *XPD*, *XPG*	Ataxia, cataracts, muscle atrophy, and neurodegeneration	Nance and Berry, 1992; Wilson et al., 2016
Trichothiodystrophy	Transcription-coupled NER	*TTDA*, *TTDN1*, *XPB*, *XPD*	Premature bone marrow exhaustion and increased risk of cancer	Faghri et al., 2008; de Boer et al., 2002
Fanconi anemia	ICL repair	*FANCA-FANCW*	Premature bone marrow exhaustion and increased risk of cancer	Ceccaldi et al., 2016; Nalepa and Clapp, 2018
Ataxia telangiectasia	DNA damage response	*ATM*	Premature bone marrow exhaustion, diabetes, and neurodegeneration	Rothblum-Oviatt et al., 2016
Mandibular hypoplasia, deafness, progeroid features, lipodystrophy syndrome	Post-replication repair and translesion DNA synthesis	*POLD1*	Diabetes, lipodystrophy, osteoporosis, steatosis, sensory loss	Weedon et al., 2013
Ruijs-Aalfs syndrome	Protein-DNA crosslink repair	*SPRTN*	Alopecia, atherosclerosis, cataracts, diabetes, premature graying of hair, osteoporosis, sarcopenia, and increased risk of cancer	Lessel et al., 2014
Alpers-Huttenlocher syndrome	Mitochondrial DNA replication and repair	*POLG1*	Progressive neurodegeneration and liver disease	Nguyen et al., 2006

Children with Hutchinson-Gilford progeroid syndrome (HGPS) develop many features of premature aging in the first decade of life including alopecia, atherosclerosis, osteolysis, and lipodystrophy among others (Hennekam, 2006). The genetic cause for HGPS are mutations in the *LMNA* gene, which encodes critical components of the nuclear lamina (Eriksson et al., 2003; De Sandre-Giovannoli et al., 2003) causing pernicious alterations in nuclear architecture resulting in genome instability (Kudlow et al., 2007). Nestor-Guillermo progeroid syndrome, caused by mutations in the nuclear lamina gene *BANF1*, has characteristics of accelerated aging due to impaired chromatin organization (Cabanillas et al., 2011; Loi et al., 2016).

Mutations in DNA helicases are linked to multiple diseases of accelerated aging. Mutations in three of the RECQ family of helicases (BLM, RECQL4, and WRN) cause progeroid syndromes (Uchiumi et al., 2015). Mutations in *WRN*, a gene that encodes a helicase responsible for managing replication stress and telomere stability, cause Werner syndrome (WS) (Kudlow et al., 2007). WS patients experience growth retardation, premature hair graying, lipodystrophy, as well as premature onset of multiple age-related diseases including arteriosclerosis, cancer, type 2 diabetes mellitus, and osteoporosis (Sugimoto, 2014). Rothmund-Thomson syndrome (RTS) is caused by mutations in *RECQL4*, encoding a helicase that participates in DNA replication and repair. RTS patients experience juvenile cataracts, epidermal atrophy, and increased cancer incidence (Croteau et al., 2012; Ghosh et al., 2012). Bloom syndrome (BS) is caused by mutations in *BLM*, which encodes a RecQ helicase critical for suppressing recombination and thereby genome instability (Nguyen et al., 2014; Hanada and Hickson, 2007). The mean lifespan of BS patients is 26 years and they have premature onset of numerous age-related diseases, including cancer, diabetes, and chronic obstructive pulmonary disease (de Renty and Ellis, 2017).

NER detects and removes DNA adducts that distort the helical structure of DNA such as adducts resulting from exposure to UV light, environmental mutagens, and certain classes of cancer chemotherapeutics (Schärer, 2013). Endogenous DNA adducts repaired by NER are cyclopurines; oxidative DNA lesions believed to contribute to neurodegeneration (Brooks, 2008). NER consists of two sub-pathways that are responsible for repair of the entire nuclear genome (global genome NER) or focused on transcribed genes (transcription-coupled NER). Defects in the former cause xeroderma pigmentosum (XP) linked to mutations in seven genes *XPA-G* (Cleaver, 1968; Lehmann et al., 2011). XP is characterized by accelerated photoaging of the skin and a 10,000-fold increased risk of skin cancer. XP patients also frequently have accelerated onset of peripheral neuropathy, sensorineural deficits, cerebral atrophy, and neurodegeneration (Kraemer and DiGiovanna, 2015). Defects in transcription-coupled NER cause CS or trichothiodystrophy (TTD). CS patients have progressive growth retardation, neurodegeneration, cataracts, osteoporosis, and metabolic dysfunction similar to old age (Nance and Berry, 1992; Wilson et al., 2016). TTD patients present with CS-like features plus brittle hair and ichthyosis (Faghri et al., 2008). XFE progeroid syndrome results from mutations in *ERCC4/FANCQ* causing reduced expression of XPF-ERCC1, a heterodimeric DNA repair endonuclease required for NER, DNA ICL repair, and the repair of some DNA DSBs (Niedernhofer et al., 2006; Ahmad et al., 2008; Bhagwat et al., 2009). The sentinel XFE patient, who presented with exceptional photosensitivity and exhibited clear signs of premature aging of virtually all organ systems, lived to only 16 years of age (Niedernhofer et al., 2006).

DNA ICLs are incredibly genotoxic lesions that impair transcription and replication by compromising DNA strand separation (Clauson et al., 2013). ICLs can be caused by endogenous metabolites (e.g., by-products of lipid peroxidation) or environmental exposures (e.g., bifunctional cancer chemotherapeutics such as cisplatin). ICLs are recognized by the Fanconi anemia (FA) repair pathway in replicating cells and by NER in non-dividing cells and are removed by endonucleases in these pathways (Ceccaldi et al., 2016). Defects in ICL repair cause FA, a rare disease in which patients exhibit accelerated bone marrow failure, increased risk of, congenital defects, endocrine dysfunction and other aging features (Nalepa and Clapp, 2018).

Mutations in the *ATM*, which encodes a serine/threonine kinase activated in response to DNA damage, cause ataxia telangiectasia (AT) (Thompson, 2012; Valentin-Vega et al., 2012). AT patients develop dysphagia, dysphonia, loss of motor control, diabetes as adolescents, premature aging of the hair and skin, consistent with accelerated aging (Rothblum-Oviatt et al., 2016). Mutations in *POLD1* and *SPRTN* cause two Werner-like progeroid syndromes: mandibular hypoplasia, deafness, progeroid features, lipodystrophy syndrome, and Ruijs-Aalfs syndrome, respectively (Weedon et al., 2013; Lessel et al., 2014). Alpers-Huttenlocher syndrome, marked by progressive neurodegeneration and liver failure, is caused by mutation in the mitochondrial DNA polymerase pol ɣ (Nguyen et al., 2006). Clinically, these syndromes are distinct. The distinctions likely arise from how critical a particular DNA repair mechanism is for protecting the integrity of a specific cell type or tissue. Yet there are also tremendous commonalities in the pathophysiology of the distinct syndromes, and the commonalties largely align with changes associated with normal aging. It is humbling to recognize that the vast majority of symptoms seen in these genome instability disorders is driven by endogenous DNA damage.

It is important to note that while the focus here is on human repair-deficiency phenotypes, DNA repair and response genes are for the most part highly conserved. Murine models of these genome instability disorders largely recapitulate the human disorders and have been extremely valuable for discerning genotype:phenotype correlations (Friedberg and Meira, 2006). Studies in patient cells, yeast, and even bacteria were all essential for establishing the mechanisms of DNA repair as well signaling mechanisms in response to unrepaired DNA damage, which contributes more potently to driving aging than the damage itself.

## Iatrogenic genotoxins drive aging

The number of cancer survivors is increasing dramatically as a consequence of improved therapeutics (Bluethmann et al., 2016). Unfortunately, so is the awareness that those treated with genotoxic agents are aging rapidly (Hurria et al., 2016). Radiation and genotoxic chemotherapy are used to kill rapidly dividing cells, like tumor cells. However, they are not specific for cancer cells. All cells in the body experience genotoxic stress during therapy and this triggers signaling events and cell fate decisions that appear to accelerate aging. Cancer survivors display premature onset of frailty and multi-morbidities associated with old age, often decades earlier than expected (Killock, 2014). For example, 20% of childhood cancer survivors have ischemic heart disease or stroke by 50 years of age compared to 1% incidence for their siblings (Armenian et al., 2018). Whether DNA damage is increased by exposure to genotoxins or by genetic depletion of repair mechanisms, the consequence is the same: accelerated aging. This supports the notion that DNA damage, regardless of whether the source is endogenous or environmental, can be both the why and how aging occurs.

It is now well documented that chemotherapy with anthracycline, a DNA intercalating agent used for treating breast cancer, causes a significant increase in the expression of *p16^INK4a^* in peripheral T cells, a recognized measure of aging (Liu et al., 2009; Sanoff et al., 2014). This increased *p16^INK4a^* expression persists for nearly a year after treatment and equates to the rise in *p16^INK4a^* expression that occurs with 15 years of chronological aging (Sanoff et al., 2014). Pediatric cancer survivors have a >2.5-fold increased incidence of disabling age-related diseases compared to their siblings by 50 years of age (Armstrong et al., 2014). This includes arthritis, cardiovascular disease, kidney, and pulmonary disease. Platinum-based therapy (causing intra- and interstrand DNA crosslinks) causes a significantly increased risk of cardiovascular disease and pre-diabetes (Baker, 2013). A central tenet of aging is frailty, which is defined as disability, reduced endurance, risk of falls, and hospitalization due to chronic diseases that typically occur at the end of life (Vetrano, 2018). Ten percent of individuals ≥65 years old are frail. Remarkably this is indistinguishable from the incidence of frailty in childhood cancer survivors in their thirties (Ness et al., 2015). Performance on physical function tests (walking and grip strength) is the same for individuals >65 years of age and survivors of pediatric cancers who are decades younger (Henderson et al., 2014). The accelerated senescence and aging observed in cancer survivors specifically treated with genotoxic agents strongly supports the view that DNA damage drives aging.

## DNA damage impacts every aspect of cell biology

**Figure 2.**
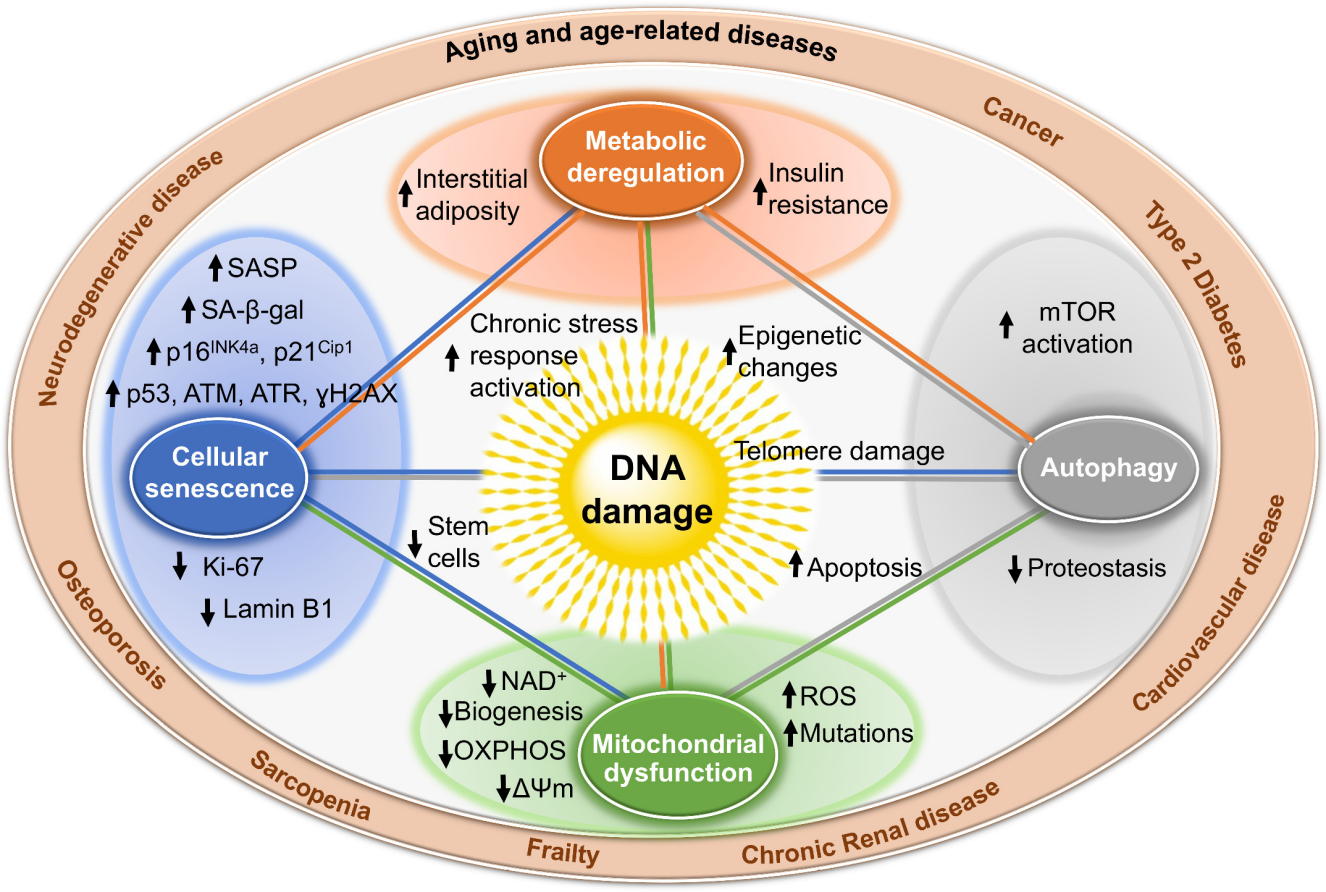
Mechanisms by which DNA damage could promote aging. DNA damage, including damage at telomeres (center), once detected, if not repaired, can interfere with replication or transcription, resulting in the activation of signaling events that alter cell physiology. One outcome of these signaling events is apoptosis, which while depleting important cells like stem cells or neurons may not be the most potent driver of aging. DNA damage can also result in mitochondrial dysfunction, impaired autophagy, metabolic changes, and the triggering of cellular senescence (small circles). These live but physiologically altered cells are predicted to be a more potent driver of aging and disease. Endpoints used to measure these consequences of DNA damage are indicated with arrows in the larger circles. These outcomes are all interconnected in that mitochondrial dysfunction can cause metabolic changes, impaired autophagy and proteostasis, more DNA damage, and senescence. This creates a cycle of increasing damage and dysfunction, which can spread to other cells via SASP, that is likely the proximal cause of aging and the diseases associated with it (outer circle).

Genotoxic stress is a potent driver of cellular senescence and senescent cells play a causal role in driving aging and age-related disease. What is truly remarkable is looking within cells harboring genotoxic stress and finding how profoundly cellular homeostasis is perturb (Figure 2). For example, in tissues of DNA repair-deficient mice (caused by reduced expression of XPF-ERCC1 due to genetic depletion of *Ercc1*), spontaneous oxidative DNA damage accumulates rapidly, albeit not to a greater extent than what occurs with aging in repair-proficient mice (Wang et al., 2012). As a consequence of this genotoxic stress, mitochondrial and metabolic dysfunction, and increased reactive oxygen species arise, identical to changes seen with normal aging (Robinson et al., 2018). Interestingly, in the same *Ercc1* mutant mice, which model XFE progeroid syndrome, senescent cells accumulate in the same organs as occurs with normal aging in mice and to roughly the same extent (Yousefzadeh et al., 2020). The profound similarities between DNA repair-deficient mice and aged mice support the notion that DNA damage drives aging. But more importantly, the cellular perturbations downstream of DNA damage reinforces ‘how’ DNA damage drives aging: by perturbing every aspect of cell biology. The consistency and uniformity with which these cellular responses to genotoxic stress occur argues that the changes are driven via signaling not as a consequence of random mutation or loss of transcription of key genes. Much remains to be elucidated about these signaling mechanisms. It is also interesting to note that the increased ROS observed in tissues of ERCC1-XPF mice is pathological and causes more DNA damage (Robinson et al., 2018), raising the point that endogenous DNA damage, if not repaired, becomes amplified.

Additional evidence to support the notion that genotoxic stress profoundly perturbs cellular homeostasis includes the following. Cells from patients with XP, CS, and AT have altered energy homeostasis, impaired mitophagy, and an increased mitochondrial membrane potential, implying accumulation of dysfunctional mitochondria and increased ATP and oxygen consumption (Valentin-Vega et al., 2012; Scheibye-Knudsen et al., 2012; Fang et al., 2014). Poly(ADP-ribose) polymerase 1 (PARP1) is critical for the detection and repair of strand breaks. Persistent activation of PARP1 depletes cellular reserves of nicotinamide adenine dinucleotide (NAD^+^) (Finkel et al., 2009), a critical co-factor for many enzymes including sirtuins, which are a family of protein deacetylases and ADP-ribosyltransferases that broadly regulate gene expression and protein stability. SIRT1 also regulates mitochondrial biogenesis by deacetylating PGC-1α (peroxisome proliferator-activated receptor γ coactivator 1 α) (Finkel et al., 2009). SIRT1 activity is dramatically reduced in animal models of XP and CS due to persistent activation of PARP1 (Scheibye-Knudsen et al., 2012; Fang et al., 2014). Inhibition of PARP1 or supplementation with NAD^+^ precursors restore SIRT1 activity and improve mitochondrial homeostasis and cellular metabolism (Scheibye-Knudsen et al., 2014; Fang et al., 2016).

## Is more DNA repair beneficial?

The gold standard for establishing a causal relationship between two events (DNA damage and aging) is to demonstrate that impaired repair accelerates aging, while improved repair slows aging. This is tricky with DNA repair as there are no drugs that stimulate DNA repair, nor is it easy to improve DNA repair genetically. DNA repair mechanisms require the coordinated action of numerous proteins. Overexpression of just one protein does not always improve repair and, in fact, can be detrimental (Shaposhnikov et al., 2015). Nevertheless, there are some hints that longevity correlates with improved responses to genotoxic stress. In the nematode *C*aenorhabditis* elegans*, 40 single gene mutations have been described that increase lifespan by at least 20% and in *all* cases these mutations confer resistance to UV irradiation (Johnson et al., 2002). Overexpression of human *MTH1*, which prevents 8-oxoG accumulation, in mice protects against neurodegeneration (De Luca et al., 2008) and extends lifespan (De Luca et al., 2013). Enhancing ATM activity in a murine model of HGPS reduces progeroid features and extends lifespan (Qian et al., 2018). Interspecies comparisons have not definitively identified a correlation between DNA repair capacity and lifespan (Austad, 2010; Cortopassi and Wang, 1996; Hart and Setlow, 1974), with a couple of exceptions. BER (but not NER) capacity is greater in cells from longer-lived rodents and non-human primates, than in shorter-lived species (Austad, 2010). Naked mole rats (lifespan 30+ years) have improved NER and BER efficiency relative to mice (lifespan 3 years) (Evdokimov et al., 2018). Many longer-lived species have increased expression or sequence optimization of key regulators of genome stability (Keane et al., 2015; Tian et al., 2017) leading to improved DSB repair in longer-lived mammals (Tian et al., 2019). Cells of centenarians have improved DNA repair activity and antioxidant capacity compared to non-centenarians (Chevanne et al., 2003; Franzke et al., 2015a). Calorie restriction (CR) is the most successful intervention to extend lifespan and/or health span in organisms ranging from yeast to mammals, including non-human primates. CR has been demonstrated to decrease the abundance of DNA damaging reactive oxygen species (reviewed in Pamplona and Barja, 2006), thereby reducing oxidative DNA damage (reviewed in Heydari et al., 2007). Consistent with this is the observation that CR reduces transcriptional stress in DNA repair defective mice (Vermeij et al., 2016). However, there is also evidence that CR might improve DNA repair, including BER, NER, and NHEJ (studies in rodents thoroughly reviewed in Heydari et al., 2007). In humans, there is evidence that CR (Matt et al., 2016), dietary micronutrients (Ames, 2010), chronic exercise, and improved socialization of the elderly (Franzke et al., 2015b) can enhance genome stability, which is ascribed to improved DNA repair capacity. However, more studies using robust measures of DNA repair capacity are needed. Collectively, there is abundant evidence that more DNA repair at least correlates with improved healthspan and lifespan.

## Conclusions

There is now sufficient and diverse evidence to support a cogent argument that DNA damage plays a causal role in aging. This includes environmental/iatrogenic sources of genotoxic stress as well as spontaneous/endogenous genotoxic stress. DNA damage contributes to aging via cell autonomous events such as causing apoptosis, which depletes functional cells such as neurons, and via cell non-autonomous mechanisms such as triggering senescence, which can negatively impact the function of neighboring, undamaged cells through their SASP. Downstream consequences of DNA damage impinge upon all of the other pillars of aging resulting in a state of self-perpetuating damage, which likely is the ultimate cause of aging. Despite these broad consequences of genotoxic stress, there is also evidence that these consequences can be modulated through approaches aimed at slowing aging, including caloric restriction, NAD+ supplementation, or ablating senescent cells. The field is still lacking tools to measure DNA lesions and DNA repair capacity that are accessible to the broader research community. Building such a tool kit would enable more precise determination of when (under what circumstances) and where (in what organs) DNA damage truly drives aging. It also might open new opportunities in precision medicine, enabling fine tuning of DNA damage and repair to, for example, improve tumor ablation, slow the loss of irreplaceable cells, or optimize metabolism to promote repair.
